# Disability and other identities?—how do they intersect?

**DOI:** 10.3389/fresc.2023.1200386

**Published:** 2023-08-10

**Authors:** Mary Wickenden

**Affiliations:** Institute of Development Studies, University of Sussex, Brighton, United Kingdom

**Keywords:** disability, rehabilitation, idisability, intersectionality, identity, inclusion, global development, gender

## Abstract

This paper addresses intersectionality and disability in global contexts. Disability as a category of identity is often omitted in rhetoric about intersectionality, which usually considers race and gender, with some consideration of other identities. However, disability like other identities is socially constructed, and liable to misrepresentation and is often siloed from other issues and experiences. Someone identifying as disabled may not be recognised by those around them as having other identities too. In discussions about intersectionality, a simplistic “additive” approach is common, while the shifting complexities and interactions between people's multiple identities are not considered with nuance. Disabled people may pragmatically adopt a kind of “strategic essentialism”. This allows them to claim a disabled identity and a specific dialogic space in order to gain recognition and perhaps access to support and services. However, they may prefer not to be classified in this dichotomised way because this ignores other aspects of them. Often an impairment is only of importance to the extent that it means that the person needs some reasonable adjustments in order to participate on equal basis with others. Arguably the SDGs and other global guidelines and treaties do not address disability as a significant identity sufficiently, nor recognise it as an important aspect of many people in combination with their other identities, rather than a stand-alone feature of them. When analysing the types of disadvantages that people experience, a broader more flexible approach is needed which recognises the ways in which different identities combine and influence people's experiences.

## Introduction

The concept of intersectionality has its roots in feminist scholarship and activism and arose initially to consider the interaction between race and gender, with the recognition of the particular disadvantage and oppression faced by black women in the US. Key figures in the early development of the idea are Audre Lorde (1) and Kimberle Crenshaw (2). Definitions of intersectionality usually emphasise both the interaction between different characteristics, identities or factors such as gender, race, age (including children and older people) and others and also the resulting impact on power dynamics and relationships for individuals who may be disadvantaged by being members of several oppressed groups (3, 4). Usually the influence both on people's agency and the role of structures are implicated. Although much of the early work on the concept was about gender and race, the literature now increasingly addresses people's situations when they identify with other disadvantaged groups, such as negatively marked sexualities, non-conforming genders, ethnicities, religions, social class and caste and or having impairments and associated disabled status. However, disability as a category of identity has often been omitted in rhetoric about intersectionality. Much of the work about intersectionality more broadly remains within the sphere of feminist scholarship and not much attention was paid to disability within this, with a few notable exceptions (5). It is only more recently that disability has been more visible as an identity of relevance in discussions about intersectionality (6).

Everyone has multiple identities with different statuses, and some people identify as being a member of one or more marginalised identities. Thus identity is a heterogenous and flexible or shifting phenomena for all. Additionally, there is variation and difference within categories or groups, so not all disabled people will have experienced the same level of stigma within a community. There are clearly similarities and contrasts between the types of oppression and disadvantages experienced by for example women, LGBTQI people or disabled people in different contexts. Power gradients can be relatively steep or less so between and across social and political arenas depending on the cultural context and from moment to moment.

An intersectional approach can help us to understand the situation for individuals or groups and how their lives are shaped by these interweaving factors. People's experiences and needs are influenced by the way that they are perceived, different aspects of a person may be responded to by others in either positive or negative ways. If someone is a member of several disadvantaged or marginalised groups, then their experience of exclusion may be exacerbated. However a purely additive approach is arguably reductive and simplifies what can be complex and shifting identities, where these multiple memberships can compound oppression but not necessarily in a straightforward cumulative way. Some identities may be more silenced than others for example. The labelling of someone's or a group's situation (e.g., black women who are disabled) as “double or triple disadvantaged” is thus simplistic, may be unhelpful and is potentially pathologising in itself. It implies a double (or triple) burden of sexism and ableism (and racism)., which then defies a more nuanced look at the person's situation and potentially overlooks their agency and strengths. Intersecting inequalities which result in increased exclusion for some are likely to be more a network of effects than linear. If taking aspects of identity into consideration were simple it would be easier to measure and plan interventions to reduce the negative impacts. This is clearly not the case as much of complex disadvantage is deeply ingrained in attitudes and behaviours at both individual and structural levels and is sadly common across cultures in high, middle-and-low-income countries.

An intersectionality approach can provide a useful lens to understand vulnerabilities such as poverty or exclusion and this is being done increasingly for example in designing social protection schemes (7). However, a danger is that this can be done too superficially. Recognition of the way that different marginalised identities intersect, can magnify disadvantage and so result in intensified stigma, discrimination and violation of human rights is important and if sufficiently sensitive to variation can inform interventions dramatically. Consideration of intersectionality in theoretical writing is often ironically rather narrow in its focus and assumes a limited number of combinations of identities which might be significant. Just as gender is not just about women, disability is likewise not a binary concept and neither are age or race. Other minority groups and identities which are relevant to an intersectional lens include: indigenous people, refugees, asylum seekers and people forced to migrate, people of nonnormative sexualities or genders (LGBTQI) (8, 9). Over the past decade, there have been a small but growing number of papers on how disability plays into this mix.

Every individual has a number of diverse subjectivities and their different characteristics and identities will be more or less relevant and salient from moment to moment in their lives. This may not be recognised by others, who may pay more attention to one aspect of a person than to others (e.g., acknowledging a woman's difference as disabled but not paying attention to their experience as a mother). There are many reasons why people may be marginalised and denigrated, experiencing diverse forms of oppression which can interact with each other. There are different ways not to be “normal” i.e., not a white heterosexual non-disabled man, often the dominant unmarked states in many cultures. The relational aspects will always be crucial and the way that power plays out between hierarchies, for individuals or within and between groups can either advantage and include, or disadvantage and exclude people.

“The normal/abnormal binary is profoundly interwoven into existing power and privilege. The construction of normalcy rationalises and bolsters the marginalisation and “othering” of bodies, minds, affects, and sexualities that do not fit into a particular culture's imaginary of the ordinary, everyday or acceptable. The suppression of diversity, the negation of potential futures (and presents and pasts) and notions about the im/perfectibility of the human are infused with fears about disability” (10).

## Disability as an identity

Importantly, following the social model of disability (11), and the gradual implementation of the UN Convention on the Rights of People with Disabilities (UN CRPD) (12), contemporary definitions emphasise the distinction between impairment (an individual's particular type of difference or difficulty: physical, sensory, cognitive, psychosocial and others or combinations) and disability (the way that they are responded to by society) (13). So disability is socially constructed, as are many other aspects of identity (e.g., gender and race). These labels or descriptions are easily at risk of misunderstanding. For each person the way that they do or do not self-identify as disabled is itself a complex and very individual process and may shift for each person over time, depending on their circumstances. Additionally, being labelled by others as different in particular ways and therefore earning the status disabled is fraught with potential wrong assumptions and misrepresentation. It is common for people identifying as disabled to feel that others pay more or undue attention to this aspect of them (and often in denigrating, oppressive ways) than to their other identities, about which they may be more concerned and interested. Disabled people themselves may not be keen on being classified in dichotomous ways (either disabled or not/or normal or not). These are often othering and excluding and may imply or assume that they have diminished experiences or views about “non disability” specific life aspects. Disabled people themselves may however adopt a kind of “strategic essentialism” as a pragmatic choice. This means that they consciously or unconsciously embrace the binary choice of disabled or not as a strategy when it is advantageous to do so. Often an impairment is only of importance to the extent that it means that someone needs some reasonable adjustments and accommodations in order to participate on equal basis with others. This approach then allows them to claim a disabled identity and a specific dialogic space in order to gain recognition and perhaps access to support and services, however this might be at the expense of recognition of other aspects of themselves or full membership of other groups. This might feel advantageous in some ways, for instance through appreciation or celebration of difference, but at the same time maybe potential limiting for an individual.

In contrast to the unwanted attention to impairment or disability aspects mentioned above, others' failure to acknowledge the specific access needs of disabled people which would enable them to access services and participate in community events as an equal citizen is common. Thus their engagement is often omitted from consultations (e.g., across sectors such as health, education, work, social protection and others, or when consulting particular people of particular ages such as children, youth or the elderly). Their voices are then missing in research or in participatory activities to gather the views of citizens, which aim to plan interventions or to inform policies (14, 15).

Thus disability as an identity, despite sometimes attracting unwanted attention is paradoxically also overlooked or misunderstood. Planners may not think that the views of this group are important, or indeed possible or necessary to gather. This reflects a lack of knowledge and “disability-aware” thinking in a whole range of agencies (UN bodies, INGOs, national and local governments, community organisations), although this is slowly changing. Despite the progress driven by the UN CRPD (12) and the rise of confident activism by the disability movement (Organisations of People with Disabilities OPDs at local, national and international levels), there is still a tendency to see disabled people as a separate category of person, in need of a “specialist” or even segregated approach, involving some kind of separate expertise, rather as part of the mainstream. What disabled people themselves say is needed is an attitude of acceptance of diversity, (or taking this further—appreciation or celebration), whatever people's apparent differences from a supposed “norm” (16). Additionally required is a willingness to adapt. Currently, their differences are seen as disruptive, disturbing or even disgusting and so difficult to understand. They are often the invisible, beyond othering, the other others (17).

## How disability intersects with other identities

Having said that discussion of intersectionality is in danger of reducing complex identities to stereotyped simplicities, it is perhaps useful, with caution, to explore some key issues raised in the literature about different intersecting identities. Shakespeare (18) talks about disability functioning as a “dustbin of disavowal” thus in opposition to “normal” (19). Sadly, several decades later this hegemonic “normalising” language is still very much in use. Once labelled as disabled, people's other ways of identifying seem to be erased and they are no longer be seen to belong to other identity groups, which are more “ordinary”. Someone's disabled status easily draws attention away from other aspects of them and the response of others may discourage them from engaging in discourses or activities related to their other identities (20). For example disabled women or men might not feel welcome at women's or men's groups which are not disability focussed. However conversely it can happen that a disabled person who also has a high-status identity such as being male, from a wealthy or educated family, or being a head of household, may be able to “trump” their impairment and achieve high status by leveraging their other identities. Disabled people often talk about the ontological dissonance between how they see themselves (selfhood) and the way they are seen by others (personhood) (21).

## Disability, gender and feminist perspectives

Of course gender does not mean consideration only of women and girls. However it is clear that often they, if they are disabled have significantly worse experiences than their male peers. Feminist perspectives on disability have been present in the theoretical literature on gender for some time (5, 22). To a lesser extent a gender lens has been present in the disability studies canon (23, 24), but arguably now there is increased attention as “feminist disability studies” *per se* (6, 25). Recognition of:- the ways in which disabled women's bodies are viewed, assumptions about their sexuality, expectations about their competence as wives and mothers and fulfil traditional gendered domestic roles, their participation in activities which are often gendered (e.g., caring roles—paid or unpaid) are all now gaining increased attention. However disabled women's lived realities arguably remain tough and they have to navigate a myriad of barriers to being heard and able to participate equally alongside other women. Thus in different cultural contexts, patriarchal and ableist structures may combine and conspire to prevent disabled women from accessing services and support, or achieving what they would like to.

“Disability, gender and discrimination are inextricably interlinked. One in five women globally live with a disability. Women are often at increased risk of developing a disability for reasons, including discrimination in health care and violence against women. Women with disabilities are also three times more likely to be illiterate, and two times less likely to be employed or use the internet”. (26).

Disabled women may feel that they are not welcomed in women's organisations or events. Then in a vicious circle which reinforces their otherness, this perpetuates the exclusion of their perspectives from the conversations these other women are having. Because they are invisible the fact that their perspectives are missing is not noticed. The UN CRPD address this exclusion:-

“International and national laws and policies on disability have historically neglected aspects related to women and girls with disabilities. In turn, laws and policies addressing women have traditionally ignored disability. This invisibility has perpetuated the situation of multiple and intersecting forms of discrimination against women and girls with disabilities” (27).

The way that the sexuality of women, men and those of other sexualities with disabilities are discussed is often in diminished and stereotyping ways and may suggest that disabled people are not sexual in the same way as everyone else. They are then either seen as asexual or hypersexual and have their “ordinary” sexual feelings and desires denied or exoticised (28, 29). Thus ordinary types of lives and indeed variations are denied to people who seen only through a disabled lens, rather than as ordinary citizens with a combination of identities and interests. It is as if one can only have one identity not several.

There has been much less written about disabled men and their gendered experiences, although there is some about the emasculation of men, and their culturally expected roles being denied or not recognised. Likewise scholarship about diverse sexualities in combination with disabilities has been relatively sparse (28, 30).

The rise of active and vocal organisations of people with disabilities (OPDs), both at local, national and international levels and their involvement in policy influencing, research and interventions is to be celebrated (16). However a word of caution is needed here as OPDs are often led by men, so again disabled women's, other genders' and sexual orientations' or people of minority races'/ethnicities' voices may not be well represented even within disability focussed arenas. There are organisations of people with particular combinations of identities, eg for women with disabilities, youth or particular impairments. However, sometimes OPDs can reinforce a siloed approach, if they are not proactive in engaging with range of identities and wider “mainstream” discussions and issues.

There has been research and advocacy around the intersection between gender and disability in a number of different sectors over the past two decades but an increasing number of more recent policy and practice pieces, related to different sectors for example about: education (31), gender-based violence (32), health (33), intersections with age (34), and about work (35, 36). Many implementers including both UN and government bodies and NGOs have begun to translate this body of knowledge into more applied forums and actions, although it is still common to see key documents omitting to mention a disability lens from the discussion.

It is clear that opportunities and challenges for disabled women and men echo those within the general population, where women are often disadvantaged, but arguably once someone has disability as an identity too (as well as others), then such inequalities increase and the uneven power gradients between men and women and between non-disabled and disabled people combine and play out with force. The age-old dichotomy between structure and agency, reminds us that the system often operates in ways which reinforce and drive the unequal and exclusionary experiences that people have at the individual level, where they are often denied agency.

## Disability and race

The intersection between race and disability is also worthy of specific consideration and again with caution as these constructs vary greatly for individuals (intersecting with other factors such as gender, social class, caste, education) and across cultural contexts. In settings where there is little or no racial or ethnic diversity, this may not be an issue, whereas in others where these are highly charged identity markers, steeped in historical disadvantage for some, the intersection with disability status can be shocking in its effects. Artiles (37) in the context of education research in the US describes the over-representation of disability within certain races

“The medical model fragments the individual, focusing either on race or on disability, rarely examining the interplay of race and disability with other key dimensions such as social class and gender” (p 331)

Clearly troubling patterns of identity recognition are closely linked to historical and colonial roots, which still influence perception of “the other” in many contexts where there are multiple racial groups (9). A linear approach which adopts a purely summative approach to the complexity of people's identities is unlikely to tackle stigma and marginalisation effectively (38). Additionally if one identity is given more focus and significance than others (e.g., too much or too little focus on disability), then the salience of other aspects of the person or group will be overlooked. Watermeyer and Schwartz (39) cogently layout the difficulty in South Africa where the conflation of black people being assumed to be poor and white being rich, means that disabled white people may not get sufficient recognition of their particular disadvantages. Thus their disability effectively disappears from view.

## Discussion

It seems then that people self-identifying or being labelled by others as disabled are at risk of two contrasting misunderstandings both of which can disadvantage them. Either their disabled status is “over attended to”, over-emphasised and perhaps exoticised and other aspects of their identity are overlooked, (e.g., giving too much attention to a child's disabilities—not recognising them as a “regular kid”). Or they are forgotten and under recognised and so not included when other groups of which they are a member are being considered (e.g., disabled women during women's events or disabled gay people in sexually nonnormative reflections etc). Thus a single identity is privileged over others and people's complex and shifting identities and roles are not acknowledged. This would likely result in disabled people's contribution to other struggles being overlooked.

Clearly a great deal of progress has been made at the global level with key treaties and goals providing guidance towards more inclusive and disability aware approaches. For example the UN Sustainable Development Goals (SDGs) (40, 41), provide pointers towards inclusion broadly across all the goals and to disability inclusion specifically in seven. However, general references to inclusion (rather than disability inclusion) are not strong enough, as often people are not sure who that refers to. There is still a tendency for policymakers, planners, funders and practitioners to parcel up disability issues into separate activities rather than rolling out a really comprehensively disability inclusive approach across sectors and arenas. Thus a project about disabled women, would ideally be re-imagined as work with all women that is automatically inclusive of disabled women and so on (42). There is still a lack of recognition of people's shifting and multiple identities and memberships. In order to achieve meaningful participation of all, there needs to be universal reasonable accommodation and accessibility across services and activities. Grunenfelder and Schurr (43) helpfully suggest that in development planning and practice, professionals should not shy away from the complexity of people's multiple identities but address them systematically. Otherwise for some individuals, their subject position (being seen as having one identity not several) might make them ineligible for particular interventions. This can generate various forms of segregation and exclusion.

The latter aspiration of “default” inclusion is still seen as a difficult to achieve ambition and one that will cost resources that haven't been allocated for this purpose. Both objections are true but indicate needs for major shifts in thinking. To achieve recognition and equality for all, radical transformations are needed. This implies turning towards the achievement of justice, both transformational and distributive (44). Perhaps also here it is useful to see this as a matter of epistemic justice. So the types of knowledge and experience that are prioritised and noticed do not yet value disability perspectives as valid and relevant (45). Disabled people's perspectives are not sought or valued. Inclusive research which deliberately seeks to include people with disabilities in data generation are an important part of making progress on understanding and promoting a disability aware and intersectionality sensitivity world view (46).

Arguably the SDGs and other global guidelines, treaties and related UN bodies outputs do not include disability as a significant identity sufficiently. nor recognise it as an important aspect for many individuals. Synergies across interest groups would help with advocacy and policy development. We need to build new coalitions and collaborations across groups. Intersecting inequalities need challenging in multiple ways and this is now being argued for more broadly, not just in relation to disability (47).

The World Bank are a good example of a global entity rolling out an intersectional approach, albeit focussing specifically on women and disability, but not yet including broader intersectionalities, such as disability and race, sexualities etc (48). Their toolkit mainly for internal use by

“task team leaders” (TTLs) offers them:-

“commitments relevant to women and girls with disabilities, examples of law and policy reform, and key barriers and solutions across several World Bank sectors, and it includes a checklist for TTLs to use throughout the project cycle. TTLs will benefit from the toolkit's key questions (focused on data collection, policy frameworks, and program development) and suggested indicators aimed to increase inclusion of women and girls with disabilities across WBG projects and a set of resources for additional support” (48).

See also the World Bank's disability inclusion accountability framework (49).

Attitudes to disability vary as much as attitudes to race or different genders, faiths or sexualities do, and are heavily influenced by cultural, social and political contexts. When analysing the particular types of disadvantages that people experience, a broader more flexible approach is needed which recognises the many and varied ways in which different identities combine and influence what people experience and how they are responded to. The kinds of disadvantages experienced are more complex than just being cumulative.

The persistence of marginalisation and disadvantage experienced by disabled people, whatever their other intersecting identities, suggests that there needs to be better recognition and incorporation of impairment and disability as important aspects of identity, which combine in complex ways with other characteristics. There is a danger that discussion about intersectionality can become rhetorical and theoretical but lacking in action. Shifts in these conversations towards nuanced and practical change and structured frameworks to monitor who is included and who is excluded, would help to reduce the stigma, lack of understanding and discrimination experienced by people, who amongst other identities, see themselves as disabled. Above all, everyone whatever their multiple identities needs to be equally included.

## Data Availability

The original contributions presented in the study are included in the article/Supplementary Material, further inquiries can be directed to the corresponding author.
